# A Dual-Linear Kalman Filter for Real-Time Orientation Determination System Using Low-Cost MEMS Sensors

**DOI:** 10.3390/s16020264

**Published:** 2016-02-20

**Authors:** Shengzhi Zhang, Shuai Yu, Chaojun Liu, Xuebing Yuan, Sheng Liu

**Affiliations:** 1 School of Mechanical & Engineering, Huazhong University of Science & Technology, Wuhan 430074, China; zhangshengzhi@hust.edu.cn (S.Z.); yushuai91@hust.edu.cn (S.Y.); liu_chaojun@126.com (C.L.); xuebing_yuan@hust.edu.cn (X.Y.); 2 School of Power and Mechanical Engineering, Wuhan University, Wuhan 430072, China

**Keywords:** sensor fusion, orientation determination, Kalman filter, MEMS

## Abstract

To provide a long-time reliable orientation, sensor fusion technologies are widely used to integrate available inertial sensors for the low-cost orientation estimation. In this paper, a novel dual-linear Kalman filter was designed for a multi-sensor system integrating MEMS gyros, an accelerometer, and a magnetometer. The proposed filter precludes the impacts of magnetic disturbances on the pitch and roll which the heading is subjected to. The filter can achieve robust orientation estimation for different statistical models of the sensors. The root mean square errors (RMSE) of the estimated attitude angles are reduced by 30.6% under magnetic disturbances. Owing to the reduction of system complexity achieved by smaller matrix operations, the mean total time consumption is reduced by 23.8%. Meanwhile, the separated filter offers greater flexibility for the system configuration, as it is possible to switch on or off the second stage filter to include or exclude the magnetometer compensation for the heading. Online experiments were performed on the homemade miniature orientation determination system (MODS) with the turntable. The average RMSE of estimated orientation are less than 0.4° and 1° during the static and low-dynamic tests, respectively. More realistic tests on two-wheel self-balancing vehicle driving and indoor pedestrian walking were carried out to evaluate the performance of the designed MODS when high accelerations and angular rates were introduced. Test results demonstrate that the MODS is applicable for the orientation estimation under various dynamic conditions. This paper provides a feasible alternative for low-cost orientation determination.

## 1. Introduction

Accurate orientation is essential for the locating and tracking of moving objects relative to a given frame in various kinds of applications, such as unmanned aerial vehicle (UAV) navigation [1], autonomous underwater vehicle (AUV) [2], self-driving cars [3], intelligent robots [4], wearable devices [5], and human position tracking [6,7,8] *etc.,* in military and industrial areas. Inertial sensors, such as the gyros and accelerometer make it possible to determine the orientation of moving object by measuring kinetic physical quantities (acceleration and angular rate, *etc.*) without any external additional information and other restrictions [9]. Gimbaled gyros, fiber optic gyros, and laser gyros have been successfully used to provide the high-precision movement information for the aircraft and missile systems in military and aviation industries. Given an initial assumption of the orientation, gyros can provide accurate orientation information via integrating the angular rates of the moving object numerically [10]. However, high-performance gyros are usually very expensive and bulky. Even the access to these devices may be limited. With the rapid expansions of the civilian and consumer markets, more and more micro-electro-mechanical system (MEMS)-based gyros are used for the low-precision orientation determination, benefited from their low price, small size, low-power dissipation, and relatively high reliability [11].

However, due to the inherent noise and drifts with time, there are considerable cumulative errors when only the gyros are used for the orientation determination. Various kinds of time series models are used to estimate the stochastic process of the gyro [12]. All of the published literature shows that the cumulative errors cannot be eliminated only according to the stochastic model of the gyro. Even for the extremely expensive MEMS gyros, the cumulative errors still cannot be neglected. Accordingly, it is necessary to integrate the gyro with some other sensors which there are no drifts and cumulative errors existing. Yun, *et al.* proposed a factored quaternion method for the orientation estimation [13]. Derivations of half-angle formulas were proposed in their paper. Taking advantage of the gravity decomposition, attitude angles (namely, the pitch and roll angles) are easily obtained from the accelerometer. Sequentially, the heading angle is available when the magnetometer is used. However, the gravity is coupled with the kinematic accelerations so strongly that it cannot be separated from the accelerometer outputs accurately, especially in the case of long-term high dynamics. Simultaneously, the geomagnetic field is very susceptible to the additional magnetic field induced by surrounding hard or soft magnet materials [14]. Due to the deviations among the directions of the geomagnetic field in different locations, it is not appropriate to use the magnetic data for the pitch and roll estimation. All the reasons above make the accelerometer and magnetometer play the role of aided sensors.

Nowadays, typical MEMS-based orientation systems usually consist of three single-axis gyros and an electronic compass which includes a tri-axis accelerometer and a tri-axis magnetometer [15,16,17]. It is crucial to develop appropriative embedded data fusion solutions for the orientation systems. The Kalman filter (KF) has already become the most commonly used sensor fusion technique for MEMS orientation systems. Various filter models were developed for different orientation representations, such as the direction cosine matrix, Euler angles, and quaternion. To simplify the implementation of KF, Zhu, *et al.* developed a linear KF in which the state vector was composed of the gravity and geomagnetic field in body frame [18]. A linear Kalman model was designed and the effect of the forgetting factor on the time lag was also investigated in their paper. Han and Wang proposed an Euler angle errors-based method to express errors in the local level frame rather than the body frame so as to achieve a linear KF [19]. Though the nonlinearity problem was avoided, the observation model was still faced with the singularity problem in their approach when the pitch angle gets through the area near ±π/2. Li and Wang developed an improved linear KF based on the psi-angle propagation equation [20]. The residuals of heading angle and accelerations were defined as the observation vector. In their paper, an adaptive gain is tuned for the KF according to the dynamic scale determined by the accelerometers when the system is in a high dynamic mode. However, not only the heading, but also that of the pitch and roll, would be affected if the magnetometer measurements were used with the accelerometer measurements directly. Furthermore, Sabatelli, *et al.* designed a two-stage extended Kalman filter (EKF) to calculate the attitude angles and the heading angle separately [21]. The accelerometer was used for the attitude determination in the first stage filter and the magnetometer was used for the heading correction in the second one. Due to the nonlinearity of the measurement equations in their method, high-order matrix operations lead to considerable increases in iterative computations eventually. Instead of implementing EKF, we designed an unscented Kalman filter (UKF) to obtain the high-accuracy indoor heading estimation in our previous works [22]. The UKF was deemed to be more accurate and less computationally costly than EKF, while too many trigonometric functions and Taylor expansions greatly increased the complexity of system. What is worse, the singularity problem was unavoidable in the UKF-based method.

In this paper, we focused on the requirements of a complete system for the low-cost orientation determination. A novel dual-linear Kalman filter was designed for the multi-sensor system. The kinematic models were based on the propagation equations of the local gravity and geomagnetic field in the body frame. The outputs of accelerometer and magnetometer were defined as the measured quantities in the two independent observation models. Benefited from the specific design, only the gyro and accelerometer are enough to run the first stage filter for the attitude estimation and a magnetometer could be integrated optionally if the heading is needed. Considering the different statistical models for sensor errors, the proposed filter can achieve optimal orientation estimation if the sensor statistical error is assumed to be white noise. The proposed strategy precludes the impacts of geomagnetic distortions on the pitch and roll which the heading is subjected to. The RMSE of attitude angles are reduced by 30.6% under magnetic disturbances. Meanwhile, owing to the reduction of system complexity, total time consumption of the proposed method is reduced by 23.8% than that of a standard one, which means that a higher frequency can be implemented for the update of orientation. Furthermore, the separated filter design offers greater flexibility and robustness to magnetic anomalies for the system configuration, as it is possible to switch on or off the second filter to include or exclude the magnetometer compensation for the heading. Online experiments were performed on the homemade real-time miniature orientation determination system (MODS). Furthermore, we carried out more realistic tests on the two-wheel self-balancing vehicle driving and indoor pedestrian walking to evaluate the performance of the designed multi-sensor system when high linear accelerations and angular rates were introduced. The test results demonstrated that the accuracy and stability of MODS were well guaranteed. This paper provides a feasible alternative for low-cost orientation determination.

The kinematic modeling is introduced for the multi-sensor system in Section 2. The dual-linear filter is proposed in Section 3. Noise characteristics are analyzed for the sensor models in Section 4. Hardware design is described for the MODS in Section 5. Real-time experiments performed on the homemade MODS are presented and discussed in Section 6. Finally, the forecasts are put forward for further study in Section 7.

## 2. System Modeling

**Figure 1:**
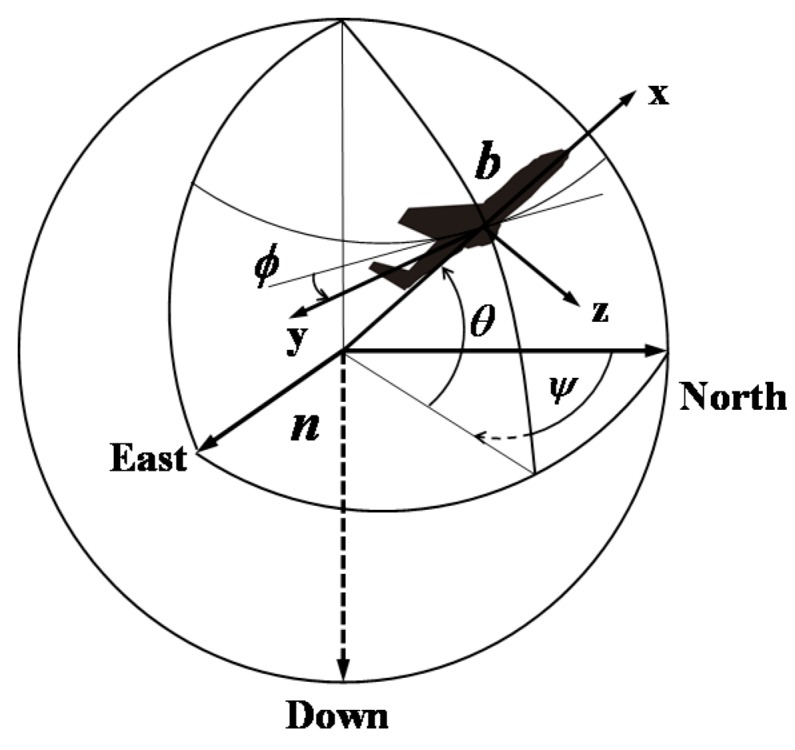
Orientation of the body frame ***b*** expressed in the navigation frame ***n***.

The orientation system is usually applied to determine Euler angles which are regarded as the essential parameters for the navigation and motion control. So-called Euler angles are defined as the rotation angles from the given inertial frame (usually called the navigation frame, denoted by ***n***) to the body frame (fixed to the moving object and denoted by ***b***), including yaw ψ, pitch θ, and roll ϕ. The body frame xyz is attached to the orientation system. The x-axis is aligned with its forward direction, the z-axis points to the bottom of the system and the y-axis rounds up the right-handed orthogonal coordinate frame. In this paper, the navigation frame ***n*** is attached to the local level north frame, namely the North, East, Down (NED) frame, as shown in Figure 1.

Here, we designate a column vector $\vec{u}$, whose components are generally functions of the time t. The transformation between the vector $\vec{u}$ in the frame ***b*** and the frame ***n*** is as:

(1) $${\vec{u}}^{n}\left(t\right)=C_{b}^{n}\left(\phi ,\theta ,\psi \right){\vec{u}}^{b}\left(t\right)$$

where $C_{b}^{n}\left(\phi ,\theta ,\psi \right)$ represents the direction cosine matrix (DCM) used to transform the measured quantities from the frame ***b*** into the frame ***n***. Henceforward, the parameter *t* will be omitted for the convenience of readers. $C_{b}^{n}$ can be carried out through three different separate rotations about the three axes. $C_{\psi }^{z}$, $C_{\theta }^{y}$, and $C_{\phi }^{x}$ represent the rotation ψ angle about the z-axis, θ angle about the y-axis, and ϕ angle about the x-axis, respectively, which are defined as:

(2) $$C_{\psi }^{z}=\left[\begin{array}{ccc} \cos\psi & -\sin\psi & 0\\ \sin\psi & \cos\psi & 0\\ 0 & 0 & 1 \end{array}\right],\;\begin{array}{c} C_{\theta }^{y}=\left[\begin{array}{ccc} \cos\theta & 0 & \sin\theta \\ 0 & 1 & 0\\ -\sin\theta & 0 & \cos\theta \end{array}\right] \end{array},\;\begin{array}{c} C_{\phi }^{x}=\left[\begin{array}{ccc} 1 & 0 & 0\\ 0 & \cos\phi & -\sin\phi \\ 0 & \sin\phi & \cos\phi \end{array}\right] \end{array}$$

Then:

(3) $$C_{b}^{n}=C_{\psi }^{z}C_{\theta }^{y}C_{\phi }^{x}=\left[\begin{array}{ccc} \cos\psi \cos\theta & \cos\psi \sin\theta \sin\phi -\sin\psi \cos\phi & \cos\psi \sin\theta \cos\phi +\sin\psi \sin\phi \\ \sin\psi \cos\theta & \sin\psi \sin\theta \sin\phi +\cos\psi \cos\phi & \sin\psi \sin\theta \cos\phi -\cos\psi \sin\phi \\ -\sin\theta & \cos\theta \sin\phi & \cos\theta \cos\phi \end{array}\right]$$

The propagation equation of $C_{b}^{n}$ accords with the following equation in [23]:

(4) $${\dot{C}}_{b}^{n}=C_{b}^{n}\cdot \left[\begin{array}{ccc} 0 & -r & q\\ r & 0 & -p\\ -q & p & 0 \end{array}\right]=C_{b}^{n}\cdot \Omega_{nb}^{b}$$

where $\Omega_{nb}^{b}$ is the skew symmetric matrix of $\omega ={\left[\begin{array}{ccc} p & q & r \end{array}\right]}^{T}$, which is the angular rate of the moving object in the frame ***b***. Differentiating Equation (1) with respect to the time:

(5) $${\dot{\vec{u}}}^{n}={\dot{C}}_{b}^{n}\cdot {\vec{u}}^{b}+C_{b}^{n}\cdot {\dot{\vec{u}}}^{b}$$

Then, the propagation equation of $\vec{u}$ in the body frame can be derived as:

(6) $${\dot{\vec{u}}}^{b}=-\Omega_{nb}^{b}\cdot {\vec{u}}^{b}$$

The vectors of the gravity and the geomagnetic field will both yield to Equation (6):

(7) $$\left\{ \begin{array}{l} {\dot{\vec{g}}}^{b}=-\Omega_{nb}^{b}\cdot {\vec{g}}^{b}\\ {\dot{\vec{m}}}^{b}=-\Omega_{nb}^{b}\cdot {\vec{m}}^{b} \end{array}\right.$$

where ${\vec{g}}^{b}={\left[\begin{array}{ccc} g_{x}^{b} & g_{y}^{b} & g_{z}^{b} \end{array}\right]}^{T}$ and ${\vec{m}}^{b}={\left[\begin{array}{ccc} m_{x}^{b} & m_{y}^{b} & m_{z}^{b} \end{array}\right]}^{T}$ represent the vectors of the gravity and the geomagnetic field in the frame ***b***, respectively.

## 3. Dual-Linear Kalman Filter Design

As discussed in the introduction, the orientation angles obtained from the accelerometer and magnetometer provide long-term accuracy with high noise while the gyro-derived orientation angles suffer from drifts and cumulative errors. Neither of them will achieve accurate and stable orientation when only one method is used alone. Therefore, a dual-linear Kalman filter is designed to integrate these two approaches together for accurate orientation estimation in this paper. Unlike Zhu *et al*. [18], defining the system state vector as $S={\left[\begin{array}{cc} {\vec{m}}^{b} & {\vec{g}}^{b} \end{array}\right]}^{T}$, we define two state vectors which are the gravity and geomagnetic field in the body frame ***b*** in this paper, respectively:

(8) $${\vec{x}}_{1}={\vec{g}}^{b},\;{\vec{x}}_{2}={\vec{m}}^{b}$$

Similarly, the accelerometer and magnetometer outputs are defined as the two observation vectors corresponding to the state vectors:

(9) $${\vec{z}}_{1}=\vec{a},\;{\vec{z}}_{2}=\vec{m}$$

The unified discrete-time dynamic equation of the system state can be expressed as follows:

(10) $$\begin{array}{l} {\vec{x}}^{n}=\Phi {\vec{x}}^{n-1}+{\vec{w}}^{n-1}\\ {\vec{z}}^{n}={\vec{x}}^{n}+{\vec{v}}^{n} \end{array}$$

where ${\vec{w}}^{n}$ and ${\vec{v}}^{n}$ are the process and measurement noises vectors, respectively. The system transfer matrix Φ is given as:

(11) $$\Phi =\text{exp}\left(-\Omega_{nb}^{b}\cdot t_{s}\right)$$

where $t_{s}$ is the sampling interval. After estimating of the state vectors, the pitch, roll, and heading are obtained by the following arc-tangent formulas:

(12) $$\left\{ \begin{array}{l} \theta =-\text{arctan}(\frac{g_{x}^{b}}{\sqrt{{\left(g_{y}^{b}\right)}^{2}+{\left(g_{z}^{b}\right)}^{2}}}),\phi =\text{arctan}(\frac{g_{y}^{b}}{g_{z}^{b}})\\ \psi =\text{arctan}(\frac{-m_{y}^{b}\cos\phi +m_{z}^{b}\sin\phi }{m_{x}^{b}\cos\theta +m_{y}^{b}\sin\phi \sin\theta +m_{z}^{b}\cos\phi \sin\theta }) \end{array}\right.$$

The next task is to compute the Kalman gain which is defined as:

(13) $$K_{n}=P_{n}^{-}H^{T}{\left(ΗP_{n}^{-}{Η}^{T}+R_{n}\right)}^{-1}$$

where $R_{n}$ is the covariance matrix of measurement noise ($R_{a}$ for the accelerometer and $R_{m}$ for the magnetometer). As the inverse of the 3 × 3 order matrix $ΗP_{n}^{-}{Η}^{T}+R_{n}$ is formulized, time consumption will be drastically reduced.

When the orientation system is in stable states, it is easy to achieve the optimal $R_{n}$. However, inertial sensor measurements may be unreliable and even useless in dynamic states. An adaptive mechanism is designed for the covariance matrixes of sensors noise in the filtering processes according to [20,24]. In the absence of magnetic disturbances, the locus of magnetometer output lies on the surface of a sphere. However, deviations of the magnetometer measurements are very large if magnetic disturbances exists. There are abundant literatures regarding the magnetometer calibration [25,26,27]. The method proposed in [28] was executed for the magnetometer calibration. The strategy will be performed for $R_{m}$ as follows:

(14) $$R_{m}=\left\{ \begin{array}{l} \sigma_{m}^{2}I,\text{if}\left|\left\|{\vec{m}}_{i+1}\right\|-\left\|\vec{h}\right\|\right|<\xi_{m}\\ \infty ,\text{otherwise} \end{array}\right.$$

**Figure 2:**
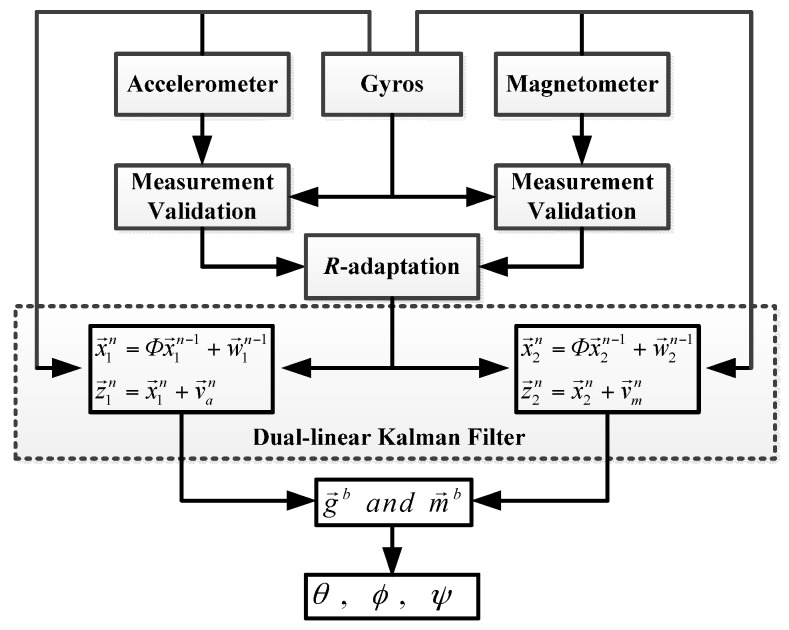
Proposed dual-linear Kalman filter.

The schematic of the proposed dual-linear filter algorithm is shown in Figure 2. The state vectors are divided into two independent linear filters and updated separately.

## 4. Noise Characteristics

The covariances of the process noise and measurement noise are regarded as the primary design parameters to achieve the minimum variance of estimation errors. In this paper, the process noise is mainly derived from the angular rates measured by the gyros. We can assume that there is a small perturbation $\delta \omega =\left[\begin{array}{ccc} \delta p & \delta q & \delta r \end{array}\right]$ to ω:

(15) $$\omega =\bar{\omega }+\delta \omega$$

where $\bar{\omega }=\left[\begin{array}{ccc} \bar{p} & \bar{q} & \bar{r} \end{array}\right]$ is the mean of ω. The state equation can be rewritten as:

(16) $$\begin{array}{l} \left[\begin{array}{c} {\dot{x}}_{1}\\ {\dot{x}}_{2}\\ {\dot{x}}_{3} \end{array}\right]=-\left[\begin{array}{ccc} 0 & -(\bar{r}+\delta r) & \left(\bar{q}+\delta q\right)\\ \left(\bar{r}+\delta r\right) & 0 & -(\bar{p}+\delta p)\\ -(\bar{q}+\delta q) & \left(\bar{p}+\delta p\right) & 0 \end{array}\right]\left[\begin{array}{c} x_{1}\\ x_{2}\\ x_{3} \end{array}\right]\\ =-\left[\begin{array}{ccc} 0 & -\bar{r} & \bar{q}\\ \bar{r} & 0 & -\bar{p}\\ -\bar{q} & \bar{p} & 0 \end{array}\right]\left[\begin{array}{c} x_{1}\\ x_{2}\\ x_{3} \end{array}\right]\underset{\vec{w}}{\underset{︸}{-\left[\begin{array}{ccc} 0 & x_{3} & -x_{2}\\ -x_{3} & 0 & x_{1}\\ x_{2} & -x_{1} & 0 \end{array}\right]\left[\begin{array}{c} \delta p\\ \delta q\\ \delta r \end{array}\right]}} \end{array}$$

The second term on the right side of the Equation (16) is regarded as the process noise $\vec{w}$. For a discrete-time system, the covariance matrix of the process noise $\vec{w}$ is obtained:

(17) $$Q=L\cdot R_{g}\cdot L^{T}$$

where

(18) $$R_{g}=diag\left[\begin{array}{ccc} \sigma_{p}^{2} & \sigma_{q}^{2} & \sigma_{r}^{2} \end{array}\right],\;L=\left[\begin{array}{ccc} 0 & x_{3} & -x_{2}\\ -x_{3} & 0 & x_{1}\\ x_{2} & -x_{1} & 0 \end{array}\right]$$

where $\sigma_{p}^{2}$, $\sigma_{q}^{2}$ and $\sigma_{r}^{2}$ are the variances of p, q, and r.

**Figure 3:**
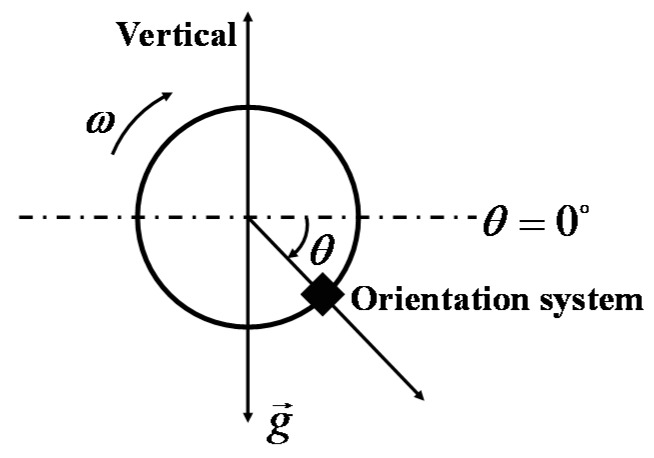
Assumed trajectory for different sensor models.

The performance of the proposed filter is strongly linked with the quality of stochastic models used to describe the different sensors’ noise. In this paper, the sensors’ noise is assumed to be a white noise according to pertinent literatures. A generalized method of wavelet moments (GMWM) proposed by Guerrier *et al*. [12] was used to analyze the impacts of different sensor error models on the filter. A numerical simulation was implemented to evaluate three different models: (1) a white noise, (2) a Gauss-Markov (GM) process, and (3) a sum of three GM processes. Firstly, we created a theoretical trajectory as shown in Figure 3. The attitude angle is expressed as follows:

(19) $$\theta =\frac{\pi }{2}\sin(0.2t)$$

where *t* is the simulation time at a sampling instant.

**Figure 4:**
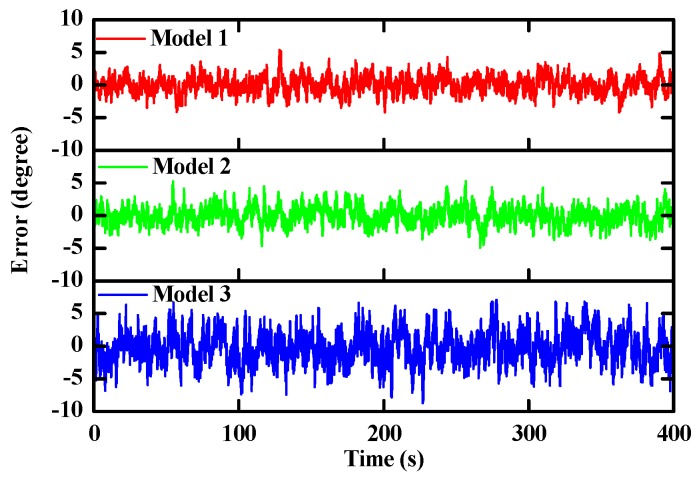
Time-varying pitch estimation errors based on three different sensor models.

**Figure 5:**
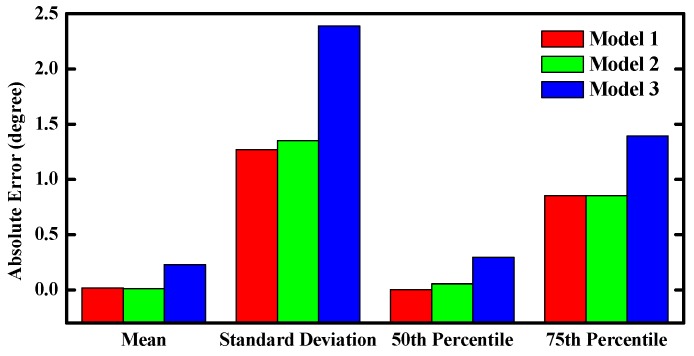
Performance comparisons of the absolute pitch estimation error.

The “perfect” inertial observations (angular rates and accelerations) were obtained by inverse strapdown. Then, we corrupted these perfect observations by three types of errors generated from the three different sensor models. Finally, the proposed filter was executed with the three kind of corrupted observations. Figure 4 shows the time-varying pitch estimation error based on three different sensor models. Figure 5 shows performance comparisons of the absolute pitch estimation error. As shown in the plots, the proposed filter can achieve robust orientation estimation for different sensors errors models. It can be seen that the proposed filter provides the optimal estimation when the sensors’ noise is assumed as a white noise. Meanwhile, the results of the Gauss-Markov model are very close to the white noise model.

## 5. Hardware Design

**Figure 6:**
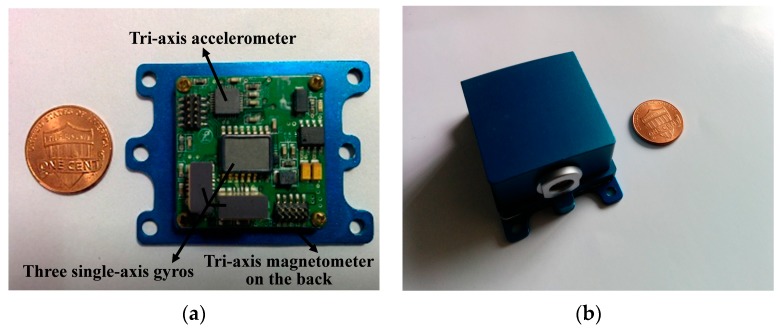
Homemade prototype of the MODS (**a**) sensors layout; (**b**) bespoke housing.

In order to verify and implement the proposed filter in practice, we developed a homemade prototype which is a real-time miniature multi-sensor system, as shown in Figure 6. The multi-sensor system is comprised of three single-axis MEMS gyros (full-scale range of ±450°/s), a tri-axis accelerometer (full-scale range of ±5 g) produced by Analog Devices, and a tri-axis magnetometer (full-scale range of ±0.8 Gauss) produced by Honeywell. Considering the electromagnetic interference, the magnetometer was welded on the back of the PCB. The sensor data fusion processes are handled by the microprocessor of the STM32F4 series for its excellent performance on dealing with large numbers of floating point arithmetic. The raw sensors data and orientation angles are collected from the miniature orientation determination system (MODS) when it is connected to the upper computer via USB or Zigbee.

## 6. Experiments and Results

### 6.1. Noise Variances Determination

**Figure 7:**
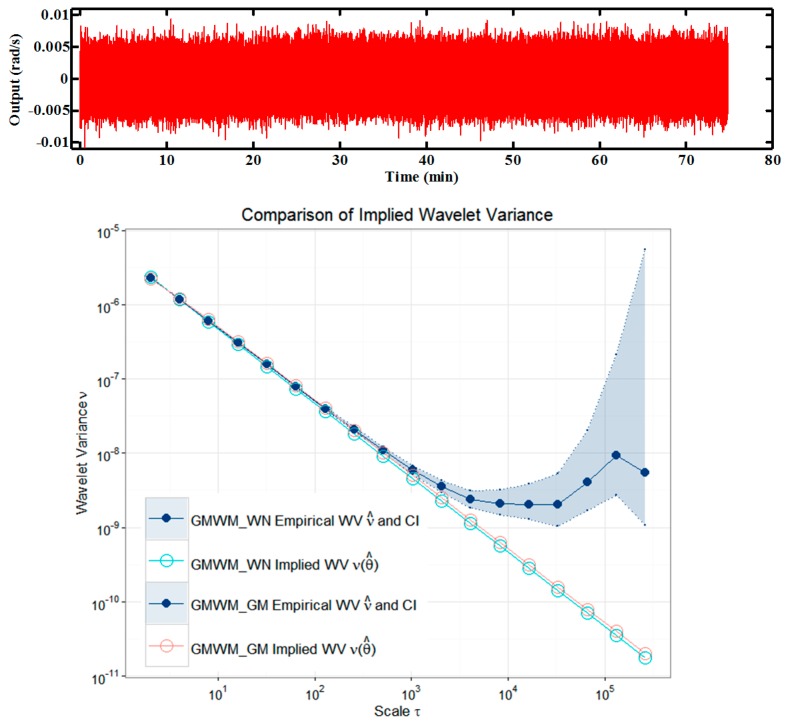
Comparisons (log–log scale) between the Haar WV and GMWM.

Firstly, we removed the mean of the original gyro signal produced in static tests at a frequency of 100 Hz (448,915 measurements). The time-varying errors are available and are presented in the time domain in Figure 7 (upper panel), together with the Haar wavelet variance (WV) and the corresponding GMWM for this process with 95% confidence intervals (lower panel). Similar results are achieved by the GMWM for a white noise process and a GM process, which validate the previous results in Section 4 again. The WV computed from the gyro signal give an indication for the underlying stochastic processes. The GMWM estimates of the parameters and their corresponding 95% confidence intervals using the WV covariances are summarized in Table 1. The suitability of the estimated models can be judged graphically by a matching of the empirical WV and the parametric WV using the estimates in Table 1.

### 6.2. Time Consumption Emulation

A time consumption test was designed to validate the effects of proposed filter for reducing the system complexity and computing time by smaller matrix operations. We collected 31,120 row data from an existing orientation system [29] at 100 Hz which were used to run our filter in MATLAB ten times, repeatedly. Table 2 shows the comparisons of time-varying pitch errors estimated by our filter (Method A) and the proposed filter in [18] (Method B). The proposed filter precludes the impacts of geomagnetic distortions on the pitch and roll which the heading is subjected to. As shown in Table 2, the average RMS errors of proposed filter are reduced by 30.6% compared with Method B under strong magnetic disturbances. Meanwhile, mean computation times are reduced by 23.8%, which means that higher frequency can be implemented for the orientation updates.

### 6.3. MODS Evaluation

Three kinds of experiments were carried out to verify the performance of the designed MODS. Firstly, the static and dynamic tests for MODS were implemented on a tri-axis turntable that allows precise and repeatable tests. Then, the MODS was mounted on a homemade two-wheel self-balancing vehicle for driving control. Finally, the MODS was tied on the shoes of pedestrian for the indoor walking and stair-climbing experiments to validate the robustness on the attitude estimation when high linear accelerations and angular rates were introduced.

#### 6.3.1. Tri-Axis Turntable Experiments for the MODS

**Figure 8:**
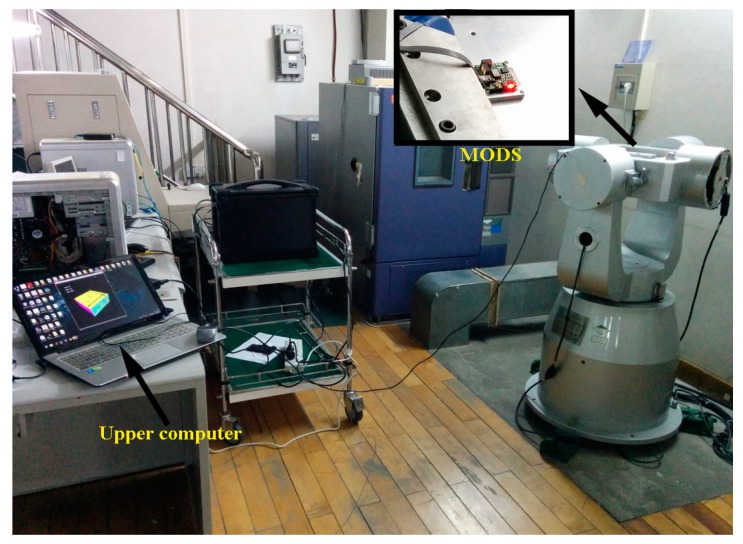
Online turntable experiments for the MODS.

As shown in Figure 8, the MODS was fixed on the static turntable relative to the ground during the static tests. In the majority of existing published literatures, orientation systems were usually kept at the zero input point so as to evaluate the static performance. In our paper, different static input points were tested in our static experiments. The attitude angles were tested from −80° to 80° and the heading was from 0° to 90°. The turntable was turned by 10° every time and would be held for a few seconds. During the dynamic test, the MODS was mounted on the running turntable which was controlled to execute the pre-set motions.

**Figure 9:**
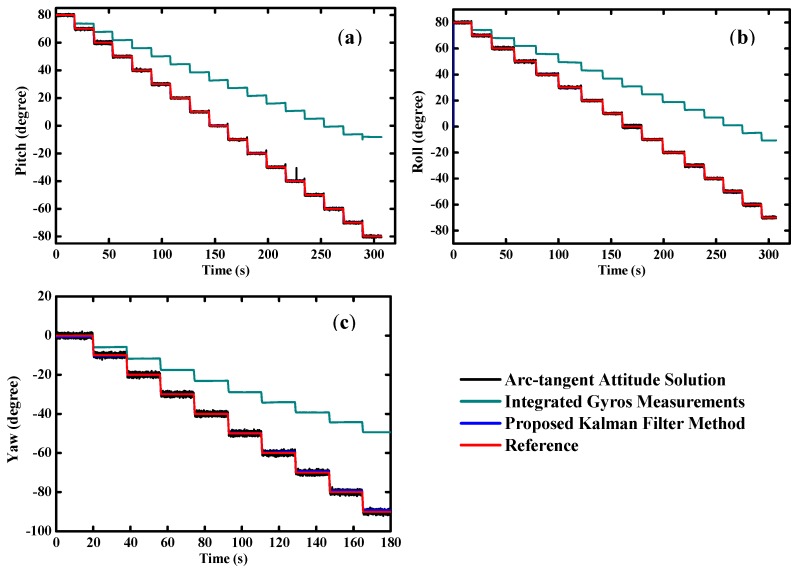
Estimated orientation during static tests (**a**) pitch angle; (**b**) roll angle; (**c**) yaw angle.

**Figure 10:**
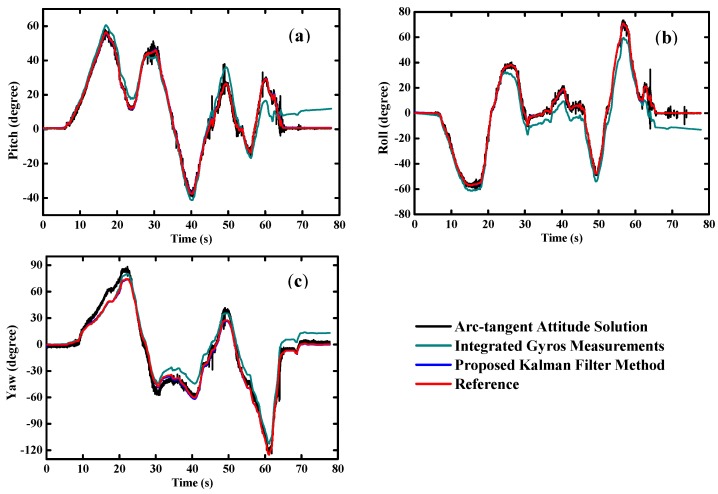
Estimated orientation during dynamic tests (**a**) pitch angle; (**b**) roll angle; (**c**) yaw angle.

As shown in Figure 9, the static test results are very smooth and steady. The dynamic test results are plotted in Figure 10. The proposed filter achieves an excellent performance on orientation estimation, which is free from the drifts of gyros and transient disturbances on the compass successfully.

Overall, the results of MODS are quite consistent with the reference, virtually without any time lag whatsoever in the tests. We can see clearly that gyro-derived orientation angles deviate from the reference due to the inherent biases of the gyros. On the other hand, the orientation angles estimated by the accelerometer and magnetometer tend to follow the reference well in the quasi-static state, but large errors and poor reliability appear in the transient high dynamic state. Table 3 indicates that the root mean square (RMS) errors of the pitch and roll angles are less than 0.1°, and approximately 0.4° for the heading angle in static tests. Meanwhile, the RMS errors are below 0.5° for the pitch and roll angles, and less than 1° for the heading angle in dynamic tests.

#### 6.3.2. Experiments on the Two-Wheel Self-Balancing Vehicle Driving

**Figure 11:**
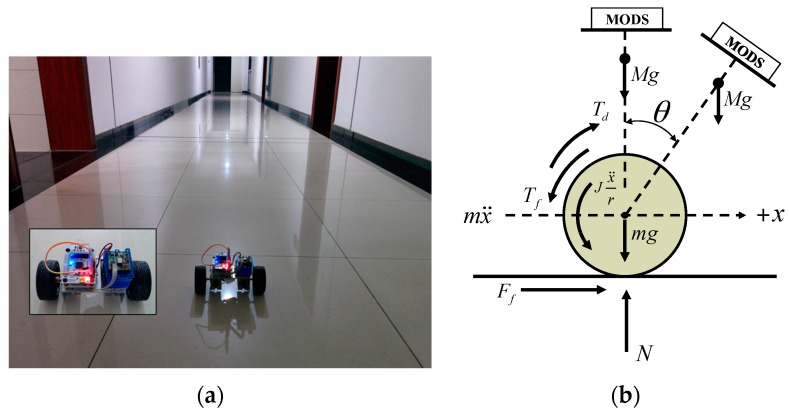
Tests on the two-wheel self-balancing vehicle (**a**) with MODS fixed on the vehicle; (**b**) schematic diagram for the vehicle driving.

**Figure 12:**
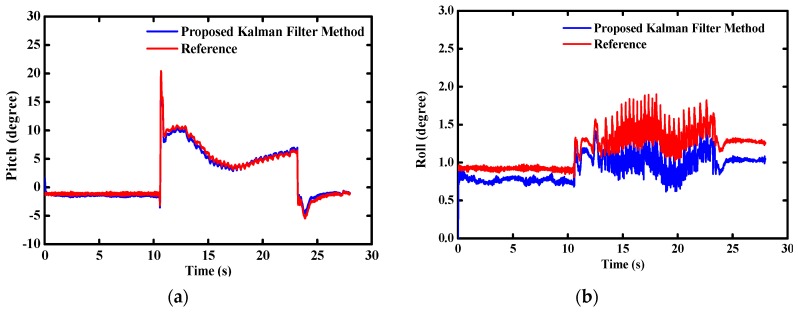
Estimated attitude angle during two-wheel self-balancing vehicle test (**a**) pitch angle; (**b**) roll angle.

For the purpose to validate the performance of the designed MODS in practical applications, we mounted it on a homemade two-wheel self-balancing vehicle, as shown in Figure 11a. The pitch angle θ is one of the most important parameters for driving the self-balancing vehicle. The servo motors will adjust the driving torque $T_{d}$ on the wheels to keep the dynamic balance of the vehicle body according to the variation of the pitch angles, as shown in Figure 11b. At the beginning, the two-wheel vehicle was kept static for a few seconds. Then, the two-wheel vehicle was controlled to drive along the preplanned route until commanded to slow down and stop. Figure 12 shows the time-varying pitch and roll angles during the whole test.

The pitch angle changes rapidly at the time of 11 s and 23 s corresponding to the starting and stopping of the two-wheel vehicle, respectively. The variations of pitch angles are relatively stable during the vehicle running. Meanwhile, the roll angle changes stably within 1° during the whole test.

#### 6.3.3. Indoor Pedestrian Walking and Stair-Climbing Experiments

**Figure 13:**
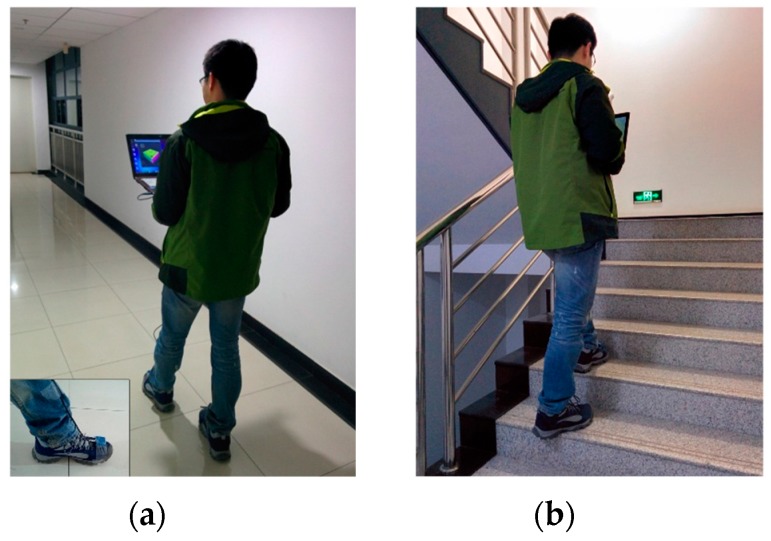
(**a**) Indoor pedestrian walking tests and (**b**) stair-climbing tests.

**Figure 14:**
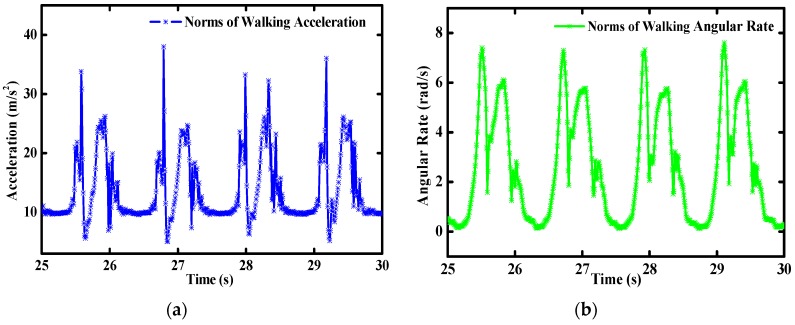
Inertial sensors measurements during walking (**a**) acceleration; (**b**) angular rate.

For the further validation of the accuracy and stability of the MODS in more realistic motions, indoor pedestrian walking and stair-climbing tests were carried out in our college building, as shown in Figure 13. The MODS was tied on the shoes of the pedestrian. During the waking tests, the pedestrian was required to walk straight along the preplanned route on the floor. The floor tiles can be regarded as the natural tags to repeat the walking tests. Raw sensor data and orientation angles were collected from the MODS. As shown in Figure 14, norms of the walking accelerations are much higher than the gravity, even close to the maximum scale range of accelerometer (5 g). Similarly, norms of walking angular rates are close to the maximum scale range of gyros (450°/s). Therefore, high linear accelerations and angular rates are introduced into the proposed filter to verify the robustness of the attitude estimation. The walking cycles are shown clearly by identifiable peak points which can be applied to indoor pedestrian navigation and human health monitoring.

**Figure 15:**
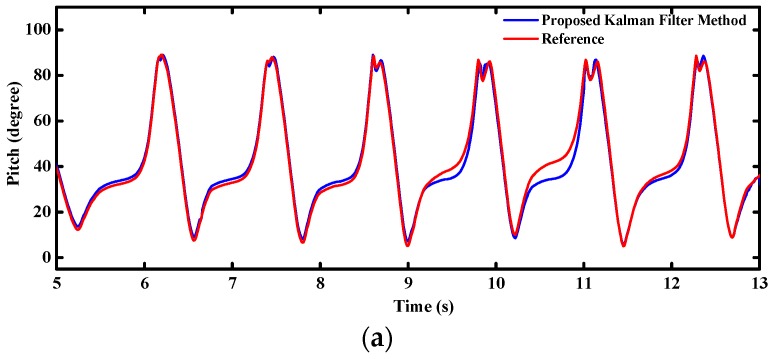

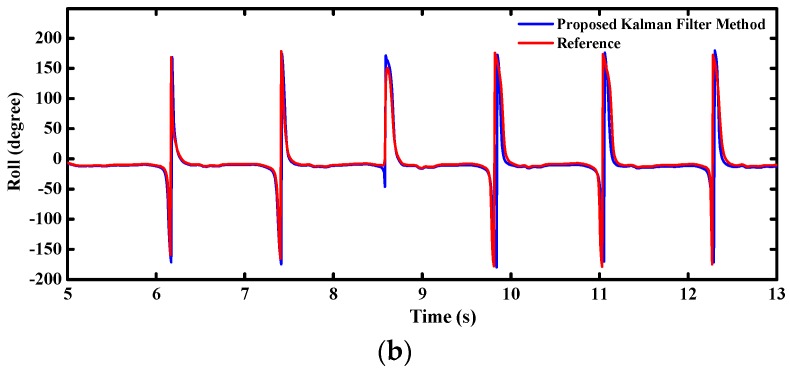
Estimated attitude angle during walking (**a**) pitch angle; (**b**) roll angle.

Figure 15 shows the real-time attitude angles of the MODS during walking tests. Similar to the variations of accelerations and angular rates in Figure 14, attitude angles change periodically with the pedestrian waking cycles. The MODS can provide stable enough attitude estimation for the pedestrian walking.

**Figure 16:**
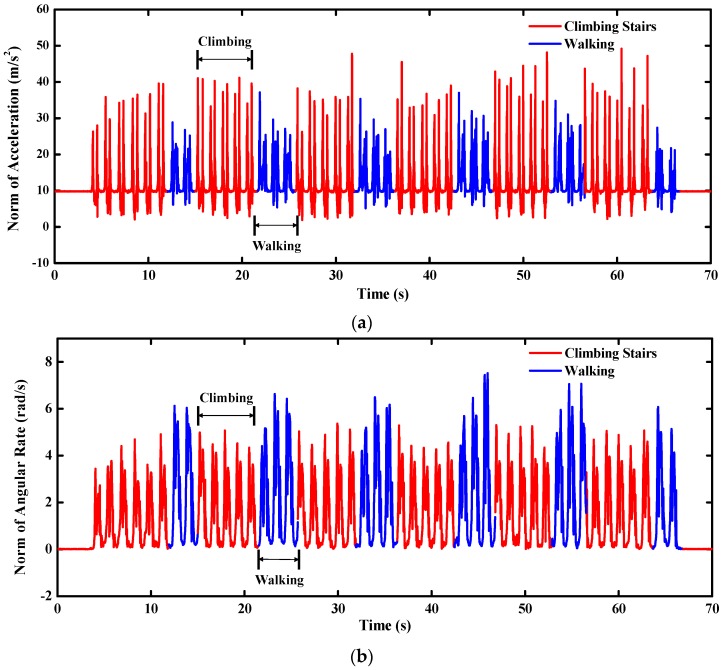
Inertial sensors measurements during stair-climbing (**a**) acceleration; (**b**) angular rate.

During the stair-climbing tests, the pedestrian was required to climb three floors including six half floors climbing and six corners walking which are corresponding to the red segments and the blue segments of the time-varying curves of the accelerations and angular rates in Figure 16. We can find out that the variation ranges of the climbing accelerations are much larger than that of the walking accelerations, but the variation ranges of the angular rates show an inverse tendency. Therefore, the proposed filter will rely more on the gyro outputs during the pedestrian walking, while the accelerometer outputs are considered more in the quasi-static attitude updates.

**Figure 17:**
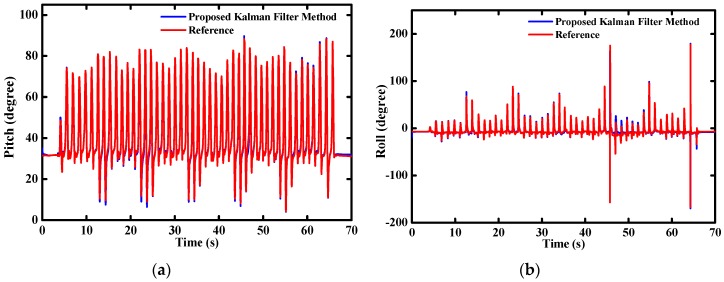
Estimated attitude angle during stair-climbing (**a**) pitch angle; (**b**) roll angle.

**Figure 18:**
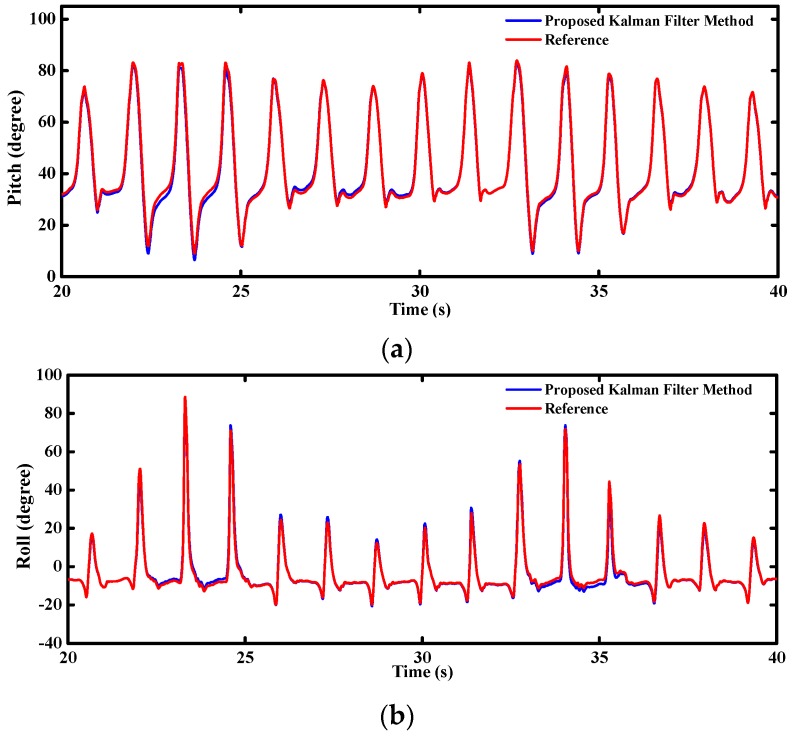
Close-ups relevant to the estimated attitude angle during stair-climbing (**a**) pitch angle; (**b**) roll angle.

Figure 17 shows the real-time attitude angles estimated by the MODS during the pedestrian stair-climbing tests. The pitch and roll angles of the walking have a larger variation range than that of climbing stairs. The results are very consistent with the realistic motions because the pedestrian’s feet can stretch completely when walking while they will be blocked by the steps when climbing stairs. Figure 18 is the close-ups relevant to the pitch and roll angles in Figure 17, which shows an excellent convergence and dynamic performance.

## 7. Conclusions

In this paper, a novel dual-linear Kalman filter was designed for the orientation determination system using low-cost MEMS-based sensors. The propagation equations of the local gravity and geomagnetic field in frame ***b*** are used to establish the dynamic models. The accelerometer and magnetometer outputs are defined as the two measured quantities in the same, but independent, observation models. The proposed strategies precludes the impacts of magnetic disturbances on the pitch and roll angles which the heading is subjected to. The RMSE of estimated attitude angles are reduced by 30.6% compared with a standard filter under magnetic disturbances. The proposed filter can achieve optimal orientation estimation if the sensor statistical error is assumed to be white noise. Owing to the reduction of system complexity, time consumption is reduced by 23.8% compared with a standard filter, which means that higher frequency can be implemented for the orientation updates. The proposed separated method offers greater flexibility for the system design. Online experiments performed on a real-time homemade MODS demonstrate that the proposed sensor fusion algorithm can maintain an accurate and stable estimation for the orientation. The average RMSE are less than 0.4° and 1° in the static and low-dynamic turntable tests, respectively. More realistic tests were carried out on the two-wheel self-balancing vehicle driving and indoor pedestrian walking to evaluate the performance of the MODS. The results demonstrate that the accuracy and stability of the MODS are guaranteed even with high linear accelerations and angular rates, which shows remarkable robustness over the applicable operating range. Therefore, the proposed approach provides a feasible alternative for the low-cost real-time orientation determination system.

Further research will focus on the calibration of MEMS devices to improve the performance of orientation determination systems. Meanwhile, some additional sensors and modules, such as the barometer and GPS, can be optionally integrated into the existing MODS. In this case, a complete orientation and position determination system would be achieved.

## Figures and Tables

**Table 1:** Estimated parameters with associated 95% confidence intervals for three different stochastic models with the gyro signal data.

Model	Parameter	Estimate	IC (0.95)
Model 1	$\sigma^{2}$	4.654064e−06	(4.639158e−06; 4.668971e−06)
Model 2	$\sigma^{2}$	4.653360e−06	(4.639986e−06; 4.666734e−06)
	β	5.342647e−02	(5.106282e−02; 5.579013e−02)
Model 3	$\sigma_{1}^{2}$	9.246705e−12	(5.989062e−12; 1.250435e−11)
	$\beta_{1}$	9.990636e−01	(9.990636e−01; 9.990636e−01)
	$\sigma_{2}^{2}$	8.794771e−13	(4.427838e−13; 1.316170e−12)
	$\beta_{2}$	9.999861e−01	(9.999861e−01; 9.999861e−01)
	$\sigma_{3}^{2}$	4.658634e−06	(4.640222e−06; 4.677046e−06)
	$\beta_{3}$	3.307696e−02	(3.307696e−02; 3.307696e−02)

**Table 2:** Comparisons of Method A and Method B.

Method	Mean Time Consumption	Mean Attitude Error
Method A	8.72 s	0.25°
Method B	11.45 s	0.36°

**Table 3:** RMS errors of the MODS in the turntable tests.

Test State	Pitch	Roll	Yaw
Static	0.045°	0.064°	0.352°
Dynamic	0.301°	0.386°	0.845°
